# Catalpol—a compound from *Rehmannia glutinosa* can improve hyperlipidemia by modulating gut microbiota and endogenous metabolic pathways

**DOI:** 10.3389/fmicb.2025.1689778

**Published:** 2025-11-11

**Authors:** Xinfeng Pei, Weichao Dong, Yingying Yu, Yinglei Wang, Shaoping Wang, Long Dai

**Affiliations:** 1 School of Pharmacy, Shandong University of Traditional Chinese Medicine, Jinan, China; 2 The Second School of Clinical Medicine, Yantai Affiliated Hospital of Binzhou Medical University, Yantai, China

**Keywords:** catalpol, hyperlipidemia, metabolomics, 16S rRNA gene sequencing, correlation analysis

## Abstract

**Introduction:**

Catalpol, an iridoid glycoside derived from *Rehmannia glutinosa*, is widely recognized for its ability to reduce blood glucose levels. However, its potential therapeutic effects on hyperlipidemia (HL) have yet to be investigated.

**Methods:**

To identify novel lipid-lowering effects of catalpol potentially exerted through the modulation of the gut microbiota and endogenous metabolic pathways, Sprague–Dawley (SD) rats were provided a high-fat diet (HFD) to induce an HL state. The lipid-lowering efficacy of catalpol was assessed using biochemical test kits. Subsequently, 16S rRNA gene sequencing was employed to analyze alterations in gut microbial composition in HL rats before and after catalpol treatment. Ultra-high-performance liquid chromatography coupled with Quadrupole Exactive Orbitrap mass spectrometry (UHPLC-Q Exactive Orbitrap MS) was used to detect and identify catalpol metabolites in plasma, urine, and feces. In addition, non-targeted metabolomics was conducted to characterize endogenous small-molecule metabolites.

**Results:**

Pharmacodynamic analysis demonstrated that catalpol markedly reduced lipid levels and inhibited hepatic lipid peroxidation. The 16S rRNA sequencing results showed that the consumption of an HFD led to a significant increase in the abundance of Firmicutes and a decrease in that of Bacteroidetes. Notably, catalpol treatment improved HL model rats’ overall gut microbiota structure. Non-targeted metabolomics revealed that the HFD significantly altered the abundance of 18 endogenous metabolites, changes that were reversed following catalpol administration. Spearman correlation analysis identified the genus *Lactobacillus* as a positive contributor to the anti-HL effect of catalpol. Furthermore, pteridine was identified as a potential biomarker associated with catalpol’s lipid-lowering activity.

**Discussion:**

Collectively, these findings demonstrate that catalpol alleviates HL by influencing gut microbiota composition and restoring plasma metabolic homeostasis.

## Graphical Abstract

**Figure:**
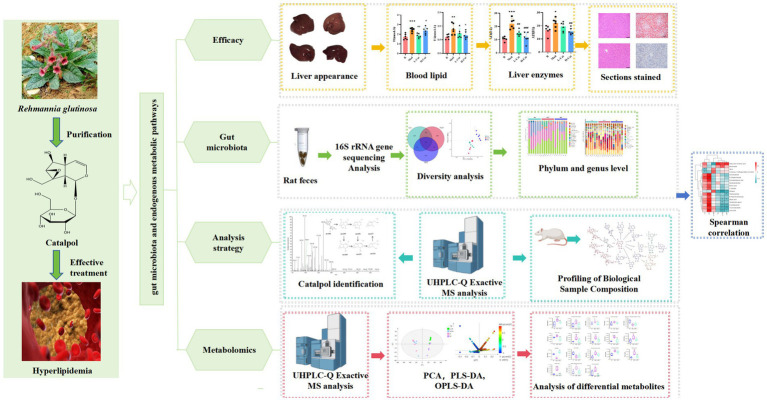

## 1 Introduction

Hyperlipidemia (HL), a common metabolic disorder, is primarily characterized by elevated plasma concentrations of total cholesterol (TC), triglycerides (TG), and low-density lipoprotein cholesterol (LDL-C) (Rosenson et al., 2024; Takaoka et al., 2024). HL is closely associated with cardiovascular and cerebrovascular diseases, notably atherosclerosis and coronary heart disease. The prevalence of HL is substantial, with 2018 national survey data indicating that it affects up to 35.6% of adults aged 18 years or older (Ahn et al., 2024; Jiang et al., 2025). The clinical management of HL relies on first-line drugs such as statins and fibrates. However, the long-term use of these drugs can lead to adverse effects, including abnormal blood glucose levels and rhabdomyolysis (Ward et al., 2019; You et al., 2025). Natural products derived from plants and animals have demonstrated significant efficacy in combating HL (Huang et al., 2019; Wu et al., 2019). The therapeutic action of iridoid glycosides in this domain is particularly noteworthy. For example, it has been shown that geniposide can reduce cholesterol accumulation and enhance its excretion via the modulation of Farnesoid X Receptor (FXR)-mediated hepatoenteric-bile acid crosstalk, thereby exerting anti-atherosclerotic effects (Liu et al., 2024). Similarly, the related compound catalpol has been shown to inhibit hepatic lipid accumulation (Huang et al., 2022).

Catalpol, an iridoid glycoside found in plants such as *Rehmannia glutinosa*, possesses multiple pharmacological properties, including antioxidant, anti-inflammatory, and hypoglycemic activities. Studies have shown that catalpol ameliorates triptolide-induced hepatic glucose metabolism disorders and oxidative stress through the modulation of the Sirtuin 1 (SIRT1)/Hypoxia-Inducible Factor-1α (HIF-1α) signaling pathway (Nie et al., 2024). However, neither the pharmacodynamic effects of catalpol on HL nor the putative underlying mechanisms have been reported to date.

The gut microbiota plays a vital role in maintaining metabolic homeostasis (Mayneris-Perxachs et al., 2021). This microbial community is often the first biological component to be disrupted during the onset and progression of metabolic diseases, leading to a metabolic imbalance within the microbial community that subsequently exacerbates systemic metabolic disorders (Du et al., 2024; Li et al., 2024). The presence of HL is closely linked to changes in the gut microbiota, characterized by an imbalance in the Firmicutes to Bacteroidetes ratio. Based on these observations, in this study, we sought to identify the mechanism by which catalpol exerts its ameliorative effects on HL from two aspects, namely, the gut microbiota and metabolites. To achieve this, Ultra-high-performance liquid chromatography coupled with Quadrupole Exactive Orbitrap mass spectrometry (UHPLC-Q-Exactive Orbitrap MS) was first employed to identify the metabolic components of catalpol (Belay et al., 2025; Ye et al., 2024). Subsequently, to determine the therapeutic effect of catalpol, changes in gut flora composition and metabolite abundance in HL model rats were evaluated through UHPLC-Q-Exactive-Orbitrap-MS-based non-targeted metabolomics and 16S rRNA gene sequencing. This study provides a basis for further exploration of the therapeutic effect of catalpol on HL. Statins primarily act by competitively inhibiting 3-hydroxy-3-methylglutaryl-coenzyme A reductase (HMG-CoA) reductase activity, thereby reducing cholesterol synthesis. However, our study demonstrates that catalpol alleviates hyperlipidemia through mechanisms involving the gut microbiota and plasma metabolites, which is distinctly different from the action of statins. Collectively, our findings reveal a novel potential role of catalpol, *Lactobacillus*, and its metabolite pteridine in HL treatment, offering new therapeutic targets and perspectives for the application of catalpol in the treatment of obesity.

## 2 Materials and methods

### 2.1 Instruments and reagents

The catalpol reference substance (purity ≥98%, verified by UV-UHPLC) was purchased from Chengdu Biopurify Phytochemicals, Ltd. (Chengdu, China, PRF24032621). Assay kits for TC, TG, high-density lipoprotein cholesterol (HDL-C), alanine aminotransferase (ALT), aspartate aminotransferase (AST), and LDL-C were obtained from Nanjing Jiancheng Bioengineering Institute (Nanjing, China).

### 2.2 Evaluation of the anti-HL effect of catalpol in rats

#### 2.2.1 Establishment of animal model

Twenty-eight male Sprague–Dawley (SD) rats, weighing 220 ± 20 g, were obtained from Jinan Pengyue Experimental Animal Breeding Co., Ltd. (Shandong, China, SYXK [RU] 2019-0003). The animal experimental procedures followed the National Institutes of Health guidelines for the care and use of experimental animals. The experimental protocol was approved by the Institutional Animal Care and Use Committee of the College of Pharmacy at Bin Zhou Medical University (Ethics Approval Number: 2025-044).

After 7 days of adaptive feeding, all the rats were randomly divided into a Blank group (*n* = 6), which received a maintenance diet, and a high-fat diet (HFD) group (*n* = 18). The HFD consisted of 5% sodium bile acid, 65% basic diet, 10% egg yolk powder, 15% lard, and 5% cholesterol (Bousette et al., 2009; González-Peña et al., 2017), supplemented with a high-fat emulsion (200 mL of water, 15 g of lard, 2.5 g of egg yolk powder, 2.5 g of cholesterol, and 0.5 g of 6-n-propyl-2-thiouracil) (Solarbio, Beijing, China). After 8 weeks of modeling, the plasma levels of TC, TG, and LDL-C in the HFD group significantly increased, while those of HDL-C significantly decreased, indicating that modeling was successful. After modeling, the HFD-fed rats were divided into three groups: a model group (Mod, *n* = 6), a low-dose catalpol (LCat) group (50 mg/kg per day catalpol), and a high-dose catalpol (HCat) group (100 mg/kg per day catalpol) (intragastric administration). Dose levels were determined in preliminary studies. The ratio of feed intake in each group is detailed in Supplementary Table S1.

#### 2.2.2 Preparation of biological samples

After being fasted for 12 h without water, all the rats were simultaneously euthanized via an intraperitoneal injection of 50 mg/kg pentobarbital sodium. Following the induction of anesthesia, blood was collected from the abdominal aorta into a 1.5 mL heparin sodium tube and centrifuged at 3,500 rpm for 15 min at 4 °C. Part of the supernatant was used for the determination of TG, TC, LDL-C, HDL-C, ALT, and AST levels using a microplate reader (SpectraMax iD5, Pleasanton, CA, USA).

### 2.3 The effect of catalpol on the gut microbiota in HFD model rats

Fecal samples were collected 1 h after the last treatment. Abdominal massage was used to promote defecation in rats. The feces were collected and placed in a 10 mL sterile tube. Three fecal pellets were collected from each rat, sealed, immediately frozen in liquid nitrogen, and stored at −80 °C.

Genomic DNA was extracted from the fecal samples using cetyl trimethyl ammonium bromide (CTAB). The V3–V4 hypervariable region of the 16S rRNA gene was PCR-amplified using the following program: pre-denaturation at 98 °C for 1 min, followed by 30 cycles of 98 °C for 10 s, 50 °C for 30 s, and 72 °C for 30 s, with a final extension at 72 °C for 5 min. Library construction was performed with the NEBNext Ultra DNA Library Prep Kit (Illumina, San Diego, CA, USA). The qualified libraries were sequenced on a NovaSeq 6000 platform.

Microbiota was carried out using PICRUSt software referencing the Kyoto Encyclopedia of Genes and Genomes (KEGG) database. Statistical Analysis of Metagenomic Profiles (STAMP) software was used for statistical analysis.

### 2.4 Identification of catalpol metabolites in rats

#### 2.4.1 Animals and drug administration

Eight male SD rats, weighing 220 ± 20 g, were obtained from Jinan Pengyue Experimental Animal Breeding Co., Ltd. (Shandong, China, SYXK [RU]2019-0003). After 1 week of acclimation, the rats were randomly divided into a Control (Blank) Group and a Catalpol group (*n* = 4/group). Before the experiment, the rats were fasted for 12 h without water. Catalpol was prepared as a suspension in normal saline. Rats in the Blank group were given 2 mL of normal saline by oral administration, while those in the Catalpol group were given 2 mL of a catalpol suspension at a dose of 300 mg/kg by oral administration.

#### 2.4.2 Sample collection and preparation

Blood samples (0.5 mL) were collected from the infraorbital venous plexus of rats at 0.5, 1, 1.5, 2, 4, and 6 h after treatment and centrifuged at 3,500 rpm for 10 min. Urine and fecal samples were collected within 24 h after oral administration. All homogeneous biological samples from the same treatment group were pooled to obtain representative group samples. Subsequently, all biological samples underwent solid-phase extraction (SPE), a method used for precipitating and concentrating proteins and solid residues. Urine, plasma, and fecal samples were added to an SPE column pretreated with methanol (3 mL) and deionized water (3 mL), respectively. Samples (1 mL) were eluted with 3 mL of deionized water, dried under nitrogen gas, and redissolved in 300 μL of methanol. After sample collection, rats were euthanized by intraperitoneal injection of pentobarbital sodium at a dose of 100 mg/kg.

#### 2.4.3 Instruments and analytical conditions

Catalpol metabolites were detected by UHPLC-Q-Exactive Orbitrap MS. Liquid chromatographic separation was performed on the DIONEX Ultimate 300 UHPLC system (Thermo Fisher Scientific, MA, USA) with the column temperature maintained at 35 °C. A 3-μL sample was injected at a 0.3 mL/min flow rate. The mobile phase consisted of 0.1% formic acid in water (solvent A) and acetonitrile (solvent B). The gradient elution program for phase B was set as follows: 0–3 min, 95–90%; 3–25 min, 90–75%; 25–30 min, 75–49%; 30–35 min, 49–35%; 35–40 min, 35–20%; 40–43 min, 20–95%; and 43–45 min, 95%.

An electrospray ionization (ESI) source was employed for detection in both positive and negative ion modes. The capillary voltage was adjusted to 35 V, and the capillary temperature was maintained at 320 °C. The auxiliary gas temperature was set to 350 °C, with 3,500 V (+) spray voltages and 3,000 V (−). The tube lens voltage was −110 V, and the sheath gas flow rate was 30 arb. Collision energies were set at 30, 45, and 60 eV. The resolution was 70,000 Full Width at Half Maximum (FWHM) for the primary scan and 17,500 FWHM for the secondary scan. The mass scan range was set between *m/z* 70 and 1,050, and the analysis was operated in full scan mode combined with data-dependent tandem mass spectrometry (MS/MS) (dd-MS^2^).

### 2.5 Non-targeted metabolomics study of catalpol against HL

#### 2.5.1 Preparation of biological samples

Four times the volume of the mixed solvent of cold methanol and acetonitrile (4,1, v/v) was added to 200 μL of plasma (plasma samples discussed in Section 2.2.2). The mixture was centrifuged at 13,000 rpm for 15 min, and the resulting supernatant was dried under nitrogen gas. The dried samples were stored at −80 °C and redissolved with 200 μL of methanol before testing.

#### 2.5.2 Data collection for UHPLC-Q-Exactive Orbitrap MS

The instrument used for analysis was the same as that described in Section 2.4.3. The column temperature was set to 40 °C. Mobile phases A and B were 0.1% formic acid in water and acetonitrile, respectively. The gradient elution conditions for phase B were as follows: 0–1 min, 5%; 1–5 min, 5–30%; 5–10 min, 30–45%; 10–15 min, 45–67%; 15–17.7 min, 67–75%; 17.7–19 min, 75–95%; 19–19.1 min, 95–5%; and 19.1–20 min, 5%. All other conditions followed the procedure described in Section 2.4.3.

#### 2.5.3 Multivariate examination using UHPLC-Q-Exactive Orbitrap MS

The initial liquid chromatography–tandem mass spectrometry (LC–MS/MS) data were processed using Thermo Fisher’s Compound Discoverer 3.0 workstation. In addition, normalization was performed to obtain a reliable dataset that included sample name, mass-to-charge ratio, mass error, signal intensity, and retention time.

In order to obtain more fragment ion peaks, we set the error range within ±5.0. At the same time, the chemical mass of the parent ion was set to the following parameters: C (0–25), H (0–40), O (0–15), S (0–4), N (0–4), and ring double bond (RDB) equivalent value (0–15).

### 2.6 Statistical analysis

Statistical analyses were performed using Statistical Package for the Social Sciences (SPSS) version 22.0 software (Chicago, IL, USA). One-way ANOVA was used for comparisons among multiple groups. Spearman correlation analysis was performed using the Wekemo Bioincloud platform^1^ (Shenzhen, China). The processed datasets were imported into Soft Independent Modelling by Class Analogy (SIMCA)-P 14.0 software (Umetrics, Sweden) for principal component analysis (PCA) and orthogonal partial least squares discriminant analysis (OPLS-DA). Differential metabolites in the OPLS-DA model were screened and identified using an S–V plot, applying a threshold of variable importance in projection (VIP) > 1 and *p* < 0.05.

## 3 Results

### 3.1 Evaluation of the efficacy of catalpol in improving HFD-induced HL rats

**Figure 1:**
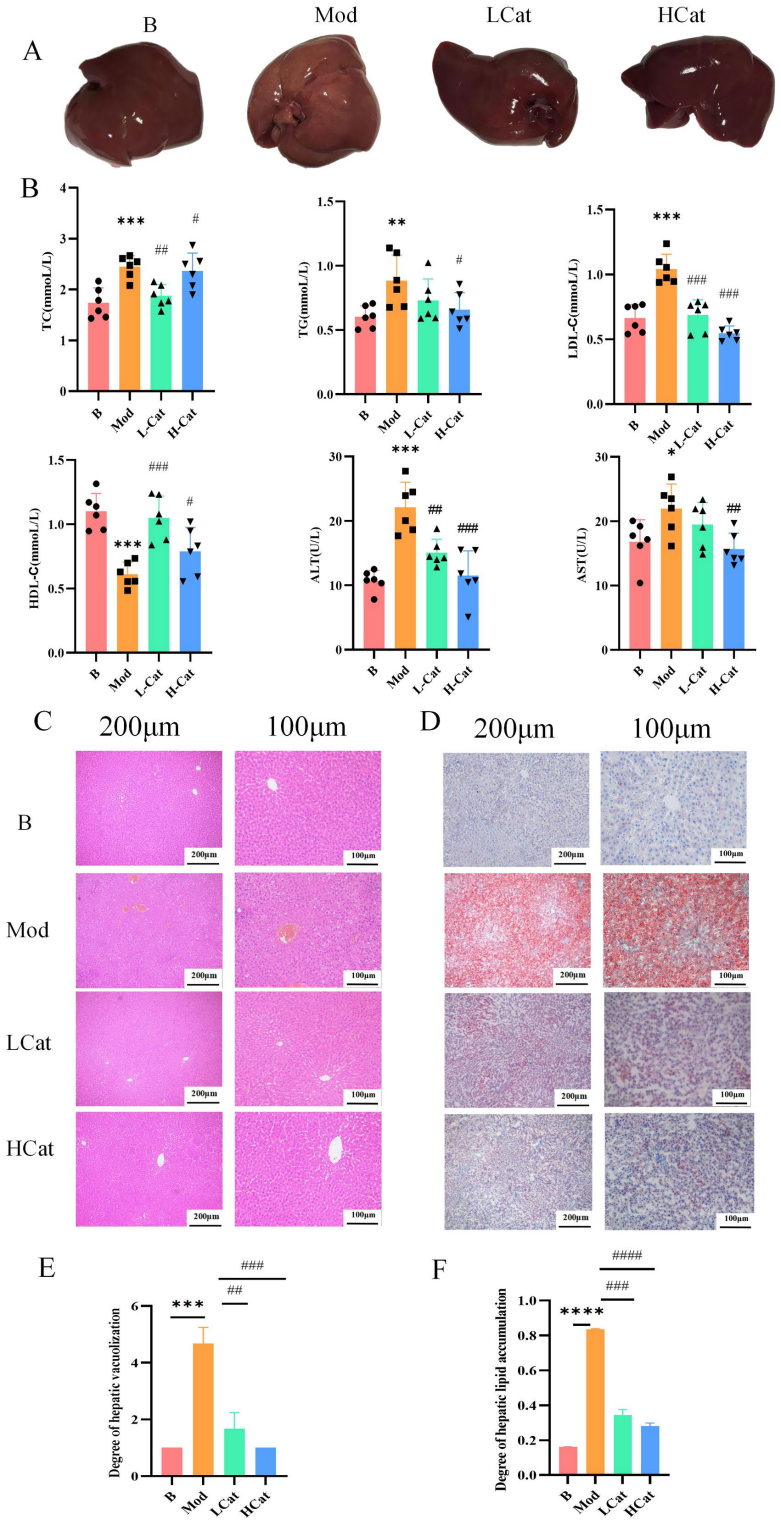
Results of the anti-HL activity of catalpol. **(A)** Appearance of the liver in the experimental rats. **(B)** Lipid-lowering effect of catalpol (TC, TG, LDL-C, HDL-C, ALT, and AST levels in rat plasma). **(C)** The results of H&E staining (200 and 100 μm). **(D)** The results of Oil Red O staining (200 and 100 μm). **(E,F)** Microscopic images were obtained from 100-μm sections stained with H&E (Left) and Oil Red O (Right). *n* = 6. ^*^p < 0.05, ^**^p < 0.01, and ^***^p < 0.001, B vs. Mod; ^#^*p* < 0.05, ^##^*p* < 0.01, and ^###^*p* < 0.001, Mod vs. H-Cat, Mod vs. L-Cat. B: Blank group; Mod: high-fat diet group; H-Cat: high-dose catalpol treatment group; L-Cat: low-dose catalpol treatment group.

The liver color of rats in the Mod group (HFD-induced HL) changed markedly compared with that of the Blank group, and the liver surfaces of the model rats were densely covered with fat particles (Figure 1A). Treatment with different doses of catalpol (LCat and HCat groups) alleviated these symptoms. Compared with the Blank group, the plasma levels of TC, TG, and LDL-C in the Mod group were significantly increased (*p* < 0.01), whereas those of HDL-C were significantly decreased (*p* < 0.01), confirming that HFD successfully induced HL in rats. In contrast, the administration of catalpol at different doses significantly reduced the levels of TC, TG, and LDL-C (*p* < 0.05) and elevated those of HDL-C (*p* < 0.05). In addition, HFD significantly increased serum ALT and AST levels (*p* < 0.05). Overall, these results suggest that catalpol has the potential to ameliorate HL (Figure 1B).

Hematoxylin and eosin (H&E) staining of the liver demonstrated hyperlipidemia-induced hepatic steatosis, characterized by the presence of intracellular lipid vacuoles in hepatocytes (Li and Wang, 2023; Liu et al., 2020). H&E staining results showed that the livers of rats in the Mod group exhibited marked steatosis and disordered hepatic cords, effects not seen in the Blank group. H&E sections also showed a significant reduction in the number of fat vacuoles following treatment. Oil Red O staining showed extensive lipid accumulation in the livers of the Mod group (Figures 1C,D). Quantitative histological scores derived from H&E and Oil Red O staining are presented in Figures 1E,F. However, treatment with different doses of catalpol partially restored hepatocyte morphology and reduced lipid deposition. Collectively, these results suggest that catalpol markedly alleviates HFD-induced hepatic lipid accumulation.

### 3.2 16S rRNA gene sequencing analysis

#### 3.2.1 Alpha diversity analysis of the gut microbiota

**Figure 2:**
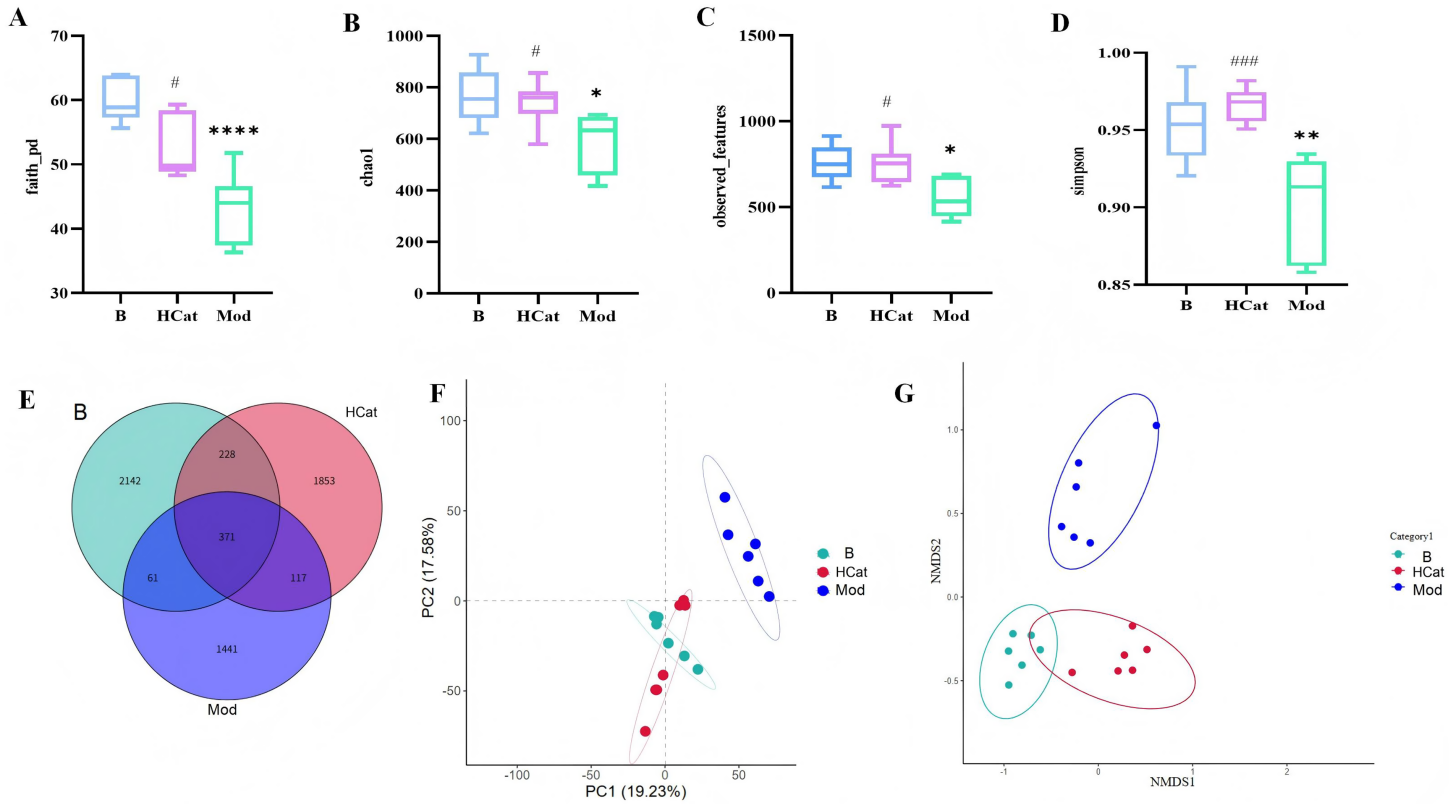
Results of 16S rRNA gene sequencing analysis. **(A–D)** Alpha diversity of gut microbiota in rats Faith’s PD index **(A)**, chao1 index **(B)**, observed features index **(C)**, Simpson index **(D)**. **(E)** Venn diagram of intestinal microflora in B, Mod, and HCat groups of bacterial community. **(F,G)** Beta diversity results of intestinal microflora in B, Mod, and HCat groups of bacterial community. PCoA analysis **(F)**; NMDS analysis **(G)**. *n* = 6. ^*^p < 0.05, ^**^p < 0.01, ^***^p < 0.001 and ^****^p < 0.0001, B vs. Mod; and ^#^*p* < 0.05, ^##^*p* < 0.01, and ^###^*p* < 0.001, Mod vs. HCat. B: blank group; Mod: high-fat diet group; HCat: catalpol high dose treatment group.

The diversity and abundance of the gut microbiota were assessed using operational taxonomic units (OTUs), Faith’s phylogenetic diversity (PD) index, Chao1 index, observed features index, and Simpson index (Figures 2A–D). The 16S rRNA sequencing results showed that all diversity indices in the Mod group exhibited a downward trend compared with those in the Blank group, indicating that the consumption of an HFD reduced the α-diversity of the gut microbiota in rats (*p* < 0.05). The significant decrease in the Simpson index reflects the heterogeneity of the microbial community.

A Venn diagram was used to compare the microbial composition across groups (Figure 2E). The results showed that the Blank and HCat groups shared the greatest number of OTUs, while the Blank and Mod groups shared the fewest. The OTU number in the Mod group was lower than in both the Blank and HCat groups. Collectively, these results suggest that an HFD induced gut microbiota dysbiosis, while high-dose catalpol administration restored the gut microbiota composition toward a normal state in rats.

#### 3.2.2 Beta diversity analysis of the gut microbiota

Principal coordinate analysis (PCoA) based on the OTU composition matrix was conducted to assess compositional differences among the samples (Figure 2F). Greater colony distances indicate larger compositional differences between microbial communities. The results showed that the Blank and Mod groups were clearly separated and distributed in distinct regions, confirming the successful establishment of the model. Non-metric multidimensional scaling (NMDS) analysis further revealed distinct clustering patterns among the three groups (Figure 2G). These findings indicate that consuming an HFD induces marked alterations in the gut microbiota of HL rats relative to normal rats, while high-dose catalpol treatment ameliorates these disturbances. This improvement may be associated with the modulatory effects of catalpol on gut microbial structure.

#### 3.2.3 Comparison of gut microbiota at the phylum and genus levels

**Figure 3:**
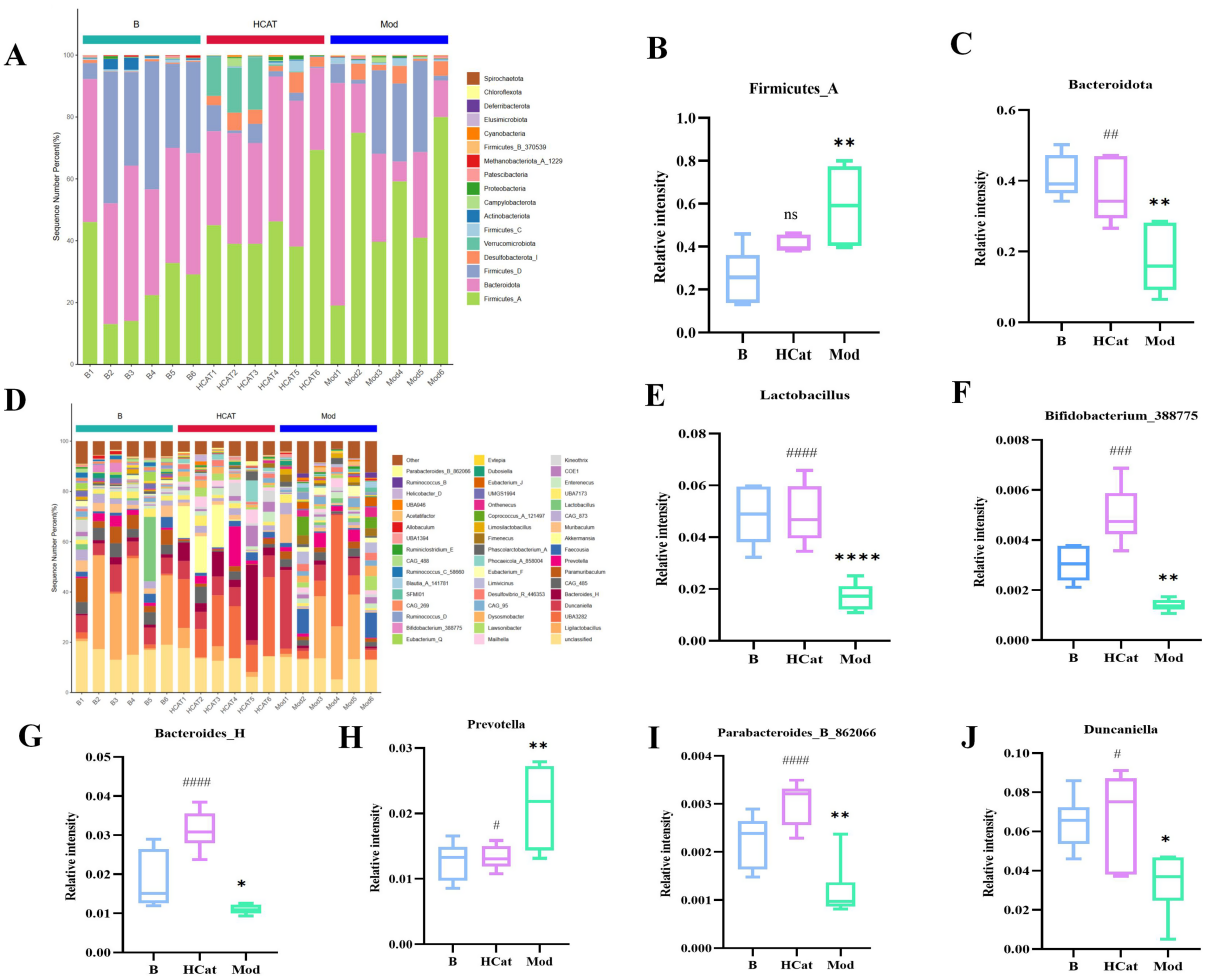
Results of 16S rRNA gene sequencing analysis. **(A)** Distribution histogram at the phylum level. **(B)** The relative abundance of Firmicutes in B, Mod, and HCat groups. **(C)** The relative abundance of Bacteroidetes in B, Mod, and HCat groups. **(D)** The distribution histogram of the genus level. Relative abundance of **(E–J)** *Lactobacillus* **(E)**, *Bifidobacterium*_388775 **(F)**, *Bacteroides*_H **(G)**, *Prevotella* **(H)**, *Parabacteroides*_B_862066 **(I)**, and *Duncaniella* **(J)**. ^*^p < 0.05, ^**^p < 0.01, ^***^p < 0.001 and ^****^p < 0.0001, B vs. Mod; and ^#^*p* < 0.05, ^##^*p* < 0.01, and ^###^*p* < 0.001, Mod vs. HCat.

A comparative analysis of microbial community composition revealed marked differences among the groups at both the phylum and genus levels. At the phylum level, the relative abundance of Bacteroidetes was significantly lower (*p* < 0.01) in the Mod group than in the Blank group, whereas that of Firmicutes was significantly higher (*p* < 0.01) (Figures 3A–C). Bacteroidetes predominated in the Blank and HCat groups, while Firmicutes were dominant in the Mod group. Treatment with catalpol effectively restored microbial balance by decreasing the abundance of Firmicutes and increasing that of Bacteroidetes, thus reversing the HFD-induced dysbiosis. The predominant genera in rat feces include *Bacteroides*, *Parabacteroides*, and *Allobaculum* (Figure 3D), among others. Consuming an HFD significantly reduced the relative abundances of *Lactobacillus*, *Bifidobacterium*_388775, *Bacteroides*_H, *Parabacteroides*_B_862066, and *Duncaniella* (*p* < 0.05), while increasing that of *Prevotella* (*p* < 0.05). Catalpol administration counteracted these changes, restoring the microbial composition toward a normal state (Figures 3E–J). These findings indicate that high-dose catalpol supplementation promotes the growth of beneficial bacterial genera, including *Lactobacillus*, *Bifidobacterium*_388775, *Bacteroides*_H, *Parabacteroides*_B_862066, and *Duncaniella*, thereby improving HFD-induced HL.

#### 3.2.4 PICRUSt2 amplicon function prediction

**Figure 4:**
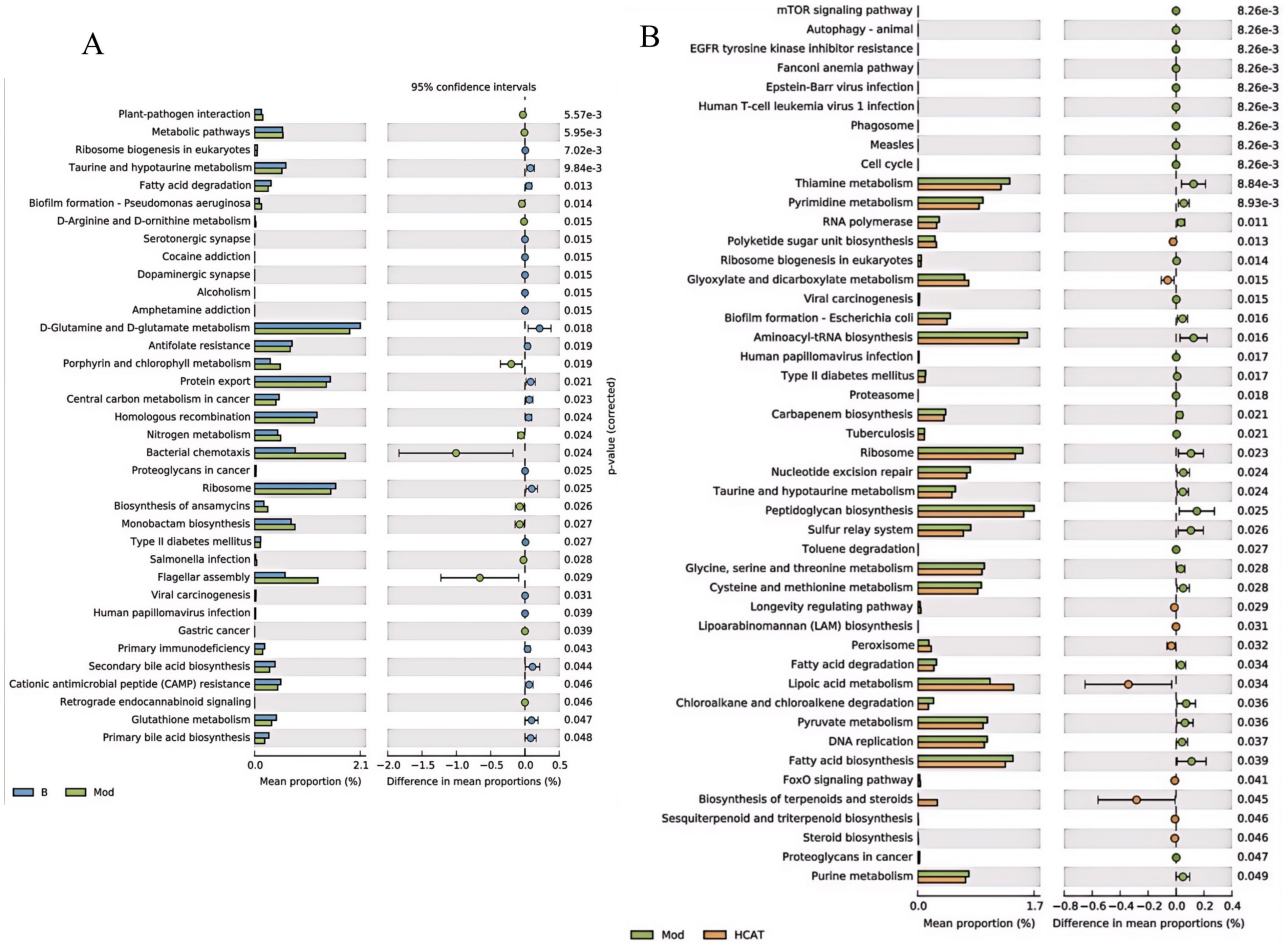
Results of PICRUSt2 amplicon function prediction. **(A)** Comparison of the function prediction results between the Blank and Model groups. **(B)** Comparison of the function prediction results between the Model and HCat groups.

Functional prediction of the gut microbiota was performed using Phylogenetic Investigation of Communities by Reconstruction of Unobserved States (PICRUSt2), and the results were analyzed with STAMP software at a significance level of *p* < 0.05. Compared with the Blank group, 36 pathways were significantly altered in the Mod group, including 13 that were upregulated and 23 that were downregulated. Notably, metabolic pathways such as fatty acid degradation, D-arginine and D-ornithine metabolism, and taurine and hypotaurine metabolism showed significant changes. Compared with the Mod group, 46 pathways were significantly altered in the HCat group, with 10 showing upregulation and 36 showing downregulation. Among them, notable alterations were observed in the mTOR signaling pathway and purine metabolism. These results indicate that high-dose catalpol can modulate the functional activity of the gut microbiota in rats given an HFD (Figure 4).

### 3.3 Catalpol responsive metabolic profiling in rats

**Figure 5:**
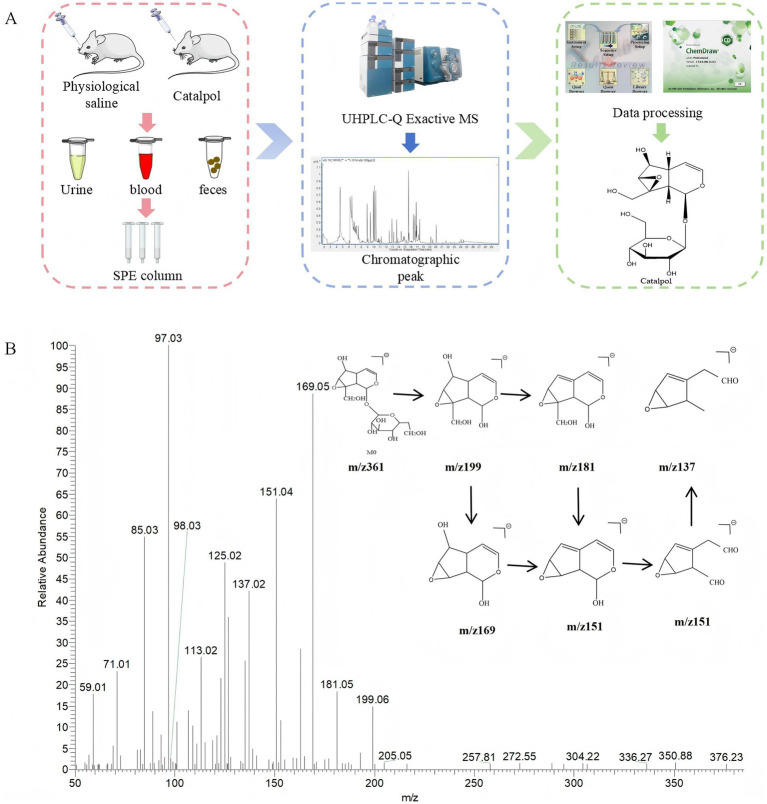
**(A)** Catalpol metabolites detection and identification analysis strategy flow chart. **(B)** The metabolic profile of catalpol.

In this study, an analytical strategy based on UHPLC–Q-Exactive Orbitrap MS was applied to investigate the metabolites of catalpol in rats (Li et al., 2023; Yi et al., 2024). Full-scan data were acquired in both positive and negative ion modes. Based on common biological reactions and structural inference, the fragmentation patterns of metabolites were proposed, and potential candidate ions were systematically identified. The overall analytical workflow is illustrated in Figure 5A.

In order to explore the metabolic process of catalpol *in vivo*, the electrospray ionization tandem mass spectrometry (ESI-MS^2^) information of catalpol standard was obtained by UHPLC-Q-Exactive MS analysis in negative ion and positive ion full scan mode. The following is the fragmentation information in negative ion mode. In this study, two drug templates were used to screen metabolites, including the structure of catalpol glycosides (*m/z* 361.11395) and catalpol aglycones (*m/z* 199.06042). The cleavage mode of catalpol was determined by identifying the redox reaction of the two template compounds mentioned above. There was a molecular ion peak (C_15_H_21_O_10_, 1.524 ppm) of catalpol at *m/z* 361.11395, and a fragment ion peak at *m/z* 199.06042 [M-H-Glc]^−^ (C_9_H_11_O_5_, 2.261 ppm); *m/z* 181.04984 [M-H-Glc-H_2_O]^−^(C_9_H_11_O_5_, 2.261 ppm); *m/z* 169.04973 [M-H-Glc-CH_2_O]^−^ (C_8_H_9_O_4_, 1.152 ppm); *m/z* 151.03902 [M-H-Glc-H_2_O-CH_2_O]^−^ (C_8_H_7_O_3_, 0.327 ppm); and *m/z* 137.02328 [M-H-Glc-H_2_O-CO_2_]^−^ (C_7_H_5_O_3_, −0.296 ppm) (Figure 5B).

### 3.4 The identification of catalpol metabolites in rats

**Figure 6:**
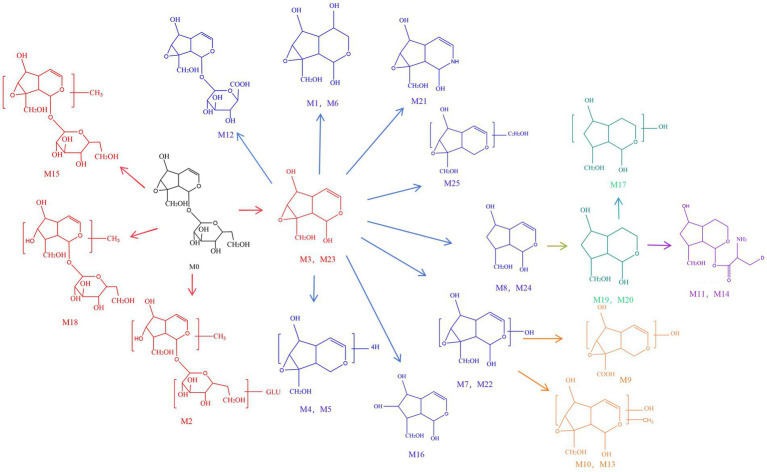
Metabolic characterization of catalpol in rats.

The UHPLC-Q-Exactive Orbitrap MS system was employed to screen and identify metabolites of catalpol in plasma, urine, and fecal samples. A total of 26 metabolites were detected (Table 1, Figure 6), 1 in positive ion mode and 25 in negative ion mode. Of the 26 metabolites, 4 were identified in plasma, 11 in urine, and 11 in feces. Additionally, three metabolites (M2, M15, and M18) were newly identified, corresponding to methylation, hydroxylation, and glycosylation products of the catalpol nucleus.

**Table 1:** Summary of identifying catalpol metabolites in rats via UHPLC-Q-Exactive Orbitrap MS.

Peaks	Product	Ion mode	Rt/min	Formula	Theoretical mass (*m/z*)	Experimental mass (*m/z*)	Error/10^−6^	MS/MS fragment ions	Source
M0	Catalpol	N	1.18	C_15_H_21_O_10_	361.11402	361.11346	1.486	97 (100), 151 (61), 137 (37), 59 (20), 71 (26), 199 (11), 181 (16)	F
M1	Hydration	N	1.23	C_9_H_13_O_6_	217.071761	217.07134	2.905	107 (100), 137 (88), 217 (47), 125 (41), 199 (7.59), 155 (7.33)	F
M2	Glycosylation	N	1.41	C_21_H_31_O_15_	523.16684	523.16711	2.606	89 (100), 199 (48), 169 (40), 137 (18)	F
M3	Deglycosylation	N	1.51	C_9_H_11_O_5_	199.06119	199.06056	2.311	125 (100), 151 (89), 181 (78), 137 (44), 199 (27)	F
M4	Didehydrogenation	N	1.72	C_9_H7O_5_	195.02989	195.02934	2.43	62 (100), 151 (2.5), 123 (1.88)	F
M5	Didehydrogenation	N	1.77	C_9_H7O_5_	195.02989	195.02934	2.77	152 (100), 196 (67), 153 (24), 167 (8), 151 (83), 123 (0.67)	P
M6	Hydration	N	1.97	C_9_H_13_O_6_	217.071761	217.07129	2.881	137 (100), 107 (98), 217 (44), 155 (11), 199 (5.9)	F
M7	Hydroxylation	N	2.4	C_9_H_11_O_6_	215.05611	215.05579	3.606	187 (100), 153 (1), 171 (1), 197 (0.94)	F
M8	Dehydroxylation and hydrogenation	N	2.48	C_9_H_13_O_4_	185.08193	185.08115	1.7	185 (100), 93 (47), 167 (5), 123 (7), 141 (5)	U
M9	Oxidation of hydroxyl to ketone carbonyl	N	4.29	C_9_H_9_O_7_	229.03537	229.02502	2.781	229 (100), 185 (36), 123 (22), 211 (7), 167 (4.13), 183 (3.6),	F
M10	Methylation and hydroxylation	N	4.29	C_10_H_13_O_6_	229.07176	229.07149	3.603	229 (100), 211 (2), 167 (1.49)	F
M11	Deglycosylation, dihydroxylation, dehydrogenation, and cysteine conjunction	N	4.41	C_12_H_20_O_6_NS	306.10168	306.10172	3.71	306 (100), 124 (86), 81 (1), 107 (0.57), 226 (0.4)	U
M12	Glucuronidation product of catalpol aglycone	N	4.48	C_16_H_23_O_10_	375.12967	375.13138	6.87	255 (100), 212 (12), 199 (1.46), 181 (0.56)	U
M13	Methylation and hydroxylation product of catalpol aglycone	N	4.59	C_10_H_13_O_6_	229.07176	229.07152	3.28	229 (100), 167 (61.1), 123 (47.42), 185 (41), 116 (37), 211 (17.57)	U
M14	Deglycosylation, dihydroxylation, dehydrogenation, and cysteine conjunction	N	4.63	C_12_H_20_O_6_NS	306.10168	306.10181	−2.79	306 (100), 124 (83), 145 (2), 81 (1), 107 (0.55), 226 (0.36)	U
M15	Methylation	N	6.87	C_16_H_23_O_10_	375.12967	375.13129	4.48	255 (100), 212 (12), 256 (4), 199 (1.46), 181 (0.74)	U
M16	Hydrogenation product	N	8.21	C_9_H_13_O_5_	201.07684	201.07614	1.94	87 (100), 201 (86), 155 (27), 121 (26), 183 (10)	F
M17	Deglycosylation, dehydroxylation, dihydrogenation, and hydroxylation	N	11.1	C_9_H_15_O_5_	203.09249	203.09221	3.988	203 (100), 185 (7), 59 (3), 139 (0.16), 155 (0.44)	U
M18	methylation and hydroxylation	N	17.55	C_16_H_25_O_9_	361.1504	361.15042	3.077	185 (100), 299 (23), 343 (3.27), 313 (1.42)	U
M19	Deglycosylation, dehydroxylation, dihydrogenation	N	14	C_9_H_15_O_4_	187.09758	187.09683	1.842	125 (100), 142 (30), 187 (16), 169 (4)	P
M20	Deglycosylation, dehydroxylation, dihydrogenation	N	14.96	C_9_H_16_O_4_	187.09758	187.09686	2.002	125 (100), 187 (80), 142 (15), 169 (2)	P
M21	Nitrogen-containing catalpol aglycone	P	15.66	C_9_H_16_O_4_N	200.09283	200.09093	−4.02	97 (100), 200 (80), 146 (33)	U
M22	Hydroxylation	N	15.74	C_9_H_11_O_6_	215.05611	215.05579	3.606	187 (100), 123 (64), 169 (33), 215 (6), 171 (1.4), 197 (1), 153 (0.83)	F
M23	Deglycosylation	N	18.84	C_9_H_11_O_5_	199.06119	199.06026	0.804	155 (100), 199 (26), 181 (15), 137 (14)	U
M24	Dehydroxylation and hydrogenation	N	23.09	C_9_H_13_O_4_	185.08193	185.08105	1.16	59 (100), 185 (21), 141 (2), 123 (0.76)	U
M25	Acetylated	N	34.81	C_11_H_13_O_6_	241.07176	241.07204	0.157	59 (100), 241 (18), 163 (10)	P

P, positive ion mode; N, negative ion mode; P, plasma samples; U, urine samples; F, feces samples.

M0 was identified as catalpol, consistent with the authentic standard. It exhibited a retention time of 1.18 min and produced the following fragment ions in negative mode electrospray ionization tandem mass spectrometry (ESI-MS^2^) mode: *m/z* 199 [M-H-Glc]^−^, 181 [M-H-Glc-H_2_O]^−^, 169 [M-H-Glc-CH_2_O]^−^, 151 [M-H-Glc-H_2_O-CH_2_O]^−^, and 137 [M-H-Glc-H_2_O-CO_2_]^−^.

M3 and M23, both 162 Da less than catalpol, were identified as catalpol aglycones and are isomeric with each other. They generated [M-H]^−^ ions at *m/z* 199.06119 (C_9_H_11_O_5_, 2.311 and 0.804 ppm) with retention times of 1.51 and 18.84 min, respectively. Analysis of their ESI-MS^2^ spectra revealed characteristic fragments at *m/z* 181 [M-H-H_2_O]^−^, 155 [M-H-CO_2_]^−^, and 137 [M-H-H_2_O-CO_2_]^−^, which confirmed their identification.

M2 was detected at a retention time of 1.41 min with an [M-H]^−^ ion at *m/z* 523.16711 (C_21_H_31_O_15_, 2.606 ppm), which is 162 Da larger than M0, suggesting a glycosylation reaction of the catalpol parent. This was confirmed by its ESI-MS^2^ spectrum, which displayed characteristic fragment ions at *m/z* 199 [M-H-2Glc]^−^, 169 [M-H-2Glc-CH_2_O]^−^, and 137 [M-H-2Glc-H_2_O-CO_2_]^−^, thereby identifying M2 as a glycosylated product of M0.

In negative ion mode, M15 (retention time: 6.87 min) exhibited a deprotonated molecular ion at *m/z* 375.13129 (C_16_H_23_O_10_, 4.48 ppm), corresponding to a mass increase of 14 Da compared to M0, which suggested a methylation reaction. This was confirmed by the presence of diagnostic fragment ions at *m/z* 199 [M-H-Glc-CH_2_]^−^ and *m/z* 181 [M-H-Glc-H_2_O-CH_2_]^−^, identifying M15 as the methylated derivative of M0.

M18, eluting at 17.55 min, exhibited a [M-H]^−^, ion at *m/z* 361.15042 (C_16_H_25_O_9_, 3.077 ppm) and produced fragment ions at *m/z* 185 [M-H-Glc]^−^, 343 [M-H-H_2_O]^−^, 299 [M-H-H_2_O-CO_2_]^−^, and 313 [M-H-H_2_O-CH_2_O]^−^. This data suggests that M18 is a hydroxylated and methylated derivative of catalpol.

As summarized in Figure 6, catalpol metabolism involves two main processes, namely, metabolism of the intact catalpol molecule and metabolism of its aglycones. Catalpol aglycones and their isomers were generated via deglycosylation, while subsequent reactions, primarily phase II biotransformations, produced a variety of metabolites. These included products of methylation, hydroxylation, glucuronidation, and glycosylation. The catalpol aglycones underwent further complex reactions such as hydroxylation, glucuronidation, methylation, hydrogenation, and dehydrogenation. Collectively, these findings provide a reference for further investigating the metabolic pathways of catalpol in rats, and further suggest that its multi-pathway metabolism may contribute to its hypolipidemic activity.

### 3.5 Plasma metabolomics analysis of the mechanism underlying the anti-HL effect of catalpol

To investigate the regulatory effect of catalpol on endogenous metabolites in rats with HFD-induced HL, the metabolic data from the Blank, Mod, LCat, and HCat groups were imported into SIMCA-P 14.0 software for comprehensive metabolomic profiling.

PCA was employed to compare metabolic profiles among the four groups (Supplementary Figures S1A,J). The clear separation observed between the Blank and Mod groups indicates that an HFD significantly altered the metabolic profile of rats. In contrast, the LCat group showed a metabolic pattern closer to that of the Mod group, while the metabolic profile of the HCat group was more similar to that of the Blank group. These results suggest that high-dose catalpol effectively restored the HFD-induced metabolic disturbances toward a normal state.

Partial least squares-discriminant analysis (PLS-DA) was further applied to validate the model’s robustness. The score plots for the Blank vs. Mod and Mod vs. HCat comparisons in both positive and negative ion modes were shown in Supplementary Figures S1B,D,K,M. The simulation coefficients of R^2^Y (0.993, 0.992) (+), R^2^Y (0.994, 0.976) (−), and Q^2^ (0.658, 0.846) (+), Q^2^ (0.848, 0.582) (−) showed the reliability of the model. The above results were also verified by 200 permutation tests (Supplementary Figures S1E,L,N).

**Figure 7:**
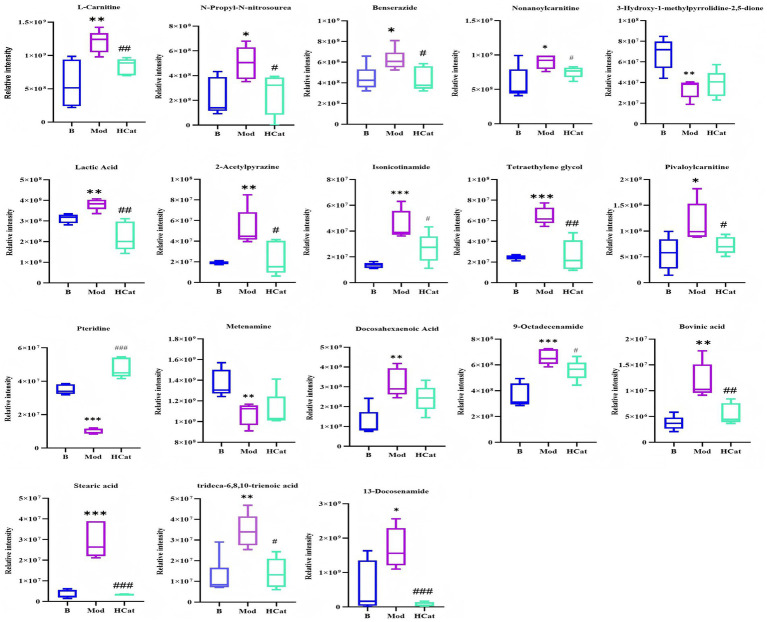
Eighteen differential metabolites were identified between the Blank and Mod groups. *n* = 6. ^*^p < 0.05, ^**^p < 0.01, and ^***^p < 0.001, B vs. Mod; and ^#^*p* < 0.05, ^##^*p* < 0.01, and ^###^*p* < 0.001, Mod vs. HCat.

OPLS-DA was employed to identify metabolites exhibiting significant differences among the experimental groups. Clear separation was observed in the OPLS-DA score plots of the Blank vs. Mod and Mod vs. HCat comparisons (Supplementary Figures S1F,H,O,Q). Differential metabolites were screened based on S-plot analysis with criteria of VIP > 1 and *p* < 0.05 (Supplementary Figures S1G,I,P,R). A total of 18 differential metabolites were identified between the Blank and Mod groups (Figure 7, Table 2). Compared with the Blank group, 15 metabolites were significantly upregulated and 3 were significantly downregulated (all *p* < 0.05) in the Mod group. Based on chemical classification, these metabolites were primarily classified as carnitines, fatty acids, heterocyclic compounds, and amides, with carnitines and fatty acids being the predominant types.

**Table 2:** Biomarkers identified as potentially regulated by catalpol.

Proposed identity	Compound	VIP	Theoretical (*m/z*)	Experimental (*m/z*)	Error (ppm)	Formula	RT	Ion mode	MS/MS fragment ions	Change trend (Mod/B)	Change trend (HCat/Mod)
L-carnitine	Amino acid analogs	7.08124	162.11246	162.11198	−3.207	C_7_H_15_NO_3_	1.503	[M + H]^+^	103 (42.25), 60 (31), 102 (14.90), 58 (19.77)	↑**	↓^##^
N-Propyl-N-nitrosourea	Nitrosoureas	4.42921	132.07675	132.07642	−2.522	C_4_H_9_N_3_O_2_	1.52	[M + H]^+^	87 (5.63), 114 (2.88), 115 (1.39)	↑*	↓^#^
Benserazide	Aromatics	4.89159	258.10844	258.10901	2.181	C_10_H_15_N_3_O_5_	1.522	[M + H]^+^	139 (100), 107 (41.31), 258 (6.48)	↑*	↓^#^
Non-anoylcarnitine	Amino acid analogs	3.03583	302.23258	302.23423	−2.129	C_16_H_31_NO_4_	1.614	[M + H]^+^	85 (100), 302 (56), 243 (8)	↑*	↓^#^
3-Hydroxy-1-methylpyrrolidine-2,5-dione	Pyrrolidinones	2.14781	128.03531	128.03398	−1.871	C_5_H7NO3	1.641	[M − H]^−^	58 (100), 42 (79.53), 83 (2.15)	↓**	↑
Lactic Acid	Carboxylic acids	5.756	88.01524	88.01291	−4.162	C_3_H_6_O_3_	1.705	[M − H]^−^	89 (100), 71 (46.84), 43 (23.52)	↑**	↓^##^
2-Acetylpyrazine	Pyrazines	1.32922	123.05528	123.05516	−1.052	C_6_H_6_N_2_O	2.62	[M + H]^+^	96 (4.04), 78 (1.85), 53 (0.73)	↑**	↓^#^
Isonicotinamide	Pyridines	2.03894	123.05528	123.0551	−1.539	C_6_H_6_N_2_O	2.975	[M + H]^+^	80 (16.26), 78 (1.85), 106 (0.95), 53 (0.73)	↑***	↓^#^
Tetraethylene glycol	Alcohols	2.4462	195.12270	195.12238	−1.641	C_8_H_18_O_5_	3.578	[M + H]^+^	89 (100), 133 (17.67), 107 (2.52)	↑***	↓^##^
Pivaloylcarnitine	Amino acid analogs	1.47363	246.16998	246.16846	4.715	C_12_H_23_NO_4_	5.021	[M + H]^+^	85 (100), 187 (8.26)	↑*	↓^#^
Pteridine	Aromatics	2.83258	131.03631	131.03496	−2.005	C_6_H_4_N_4_	5.791	[M − H]^−^	131 (100), 77 (10.18), 104 (4.51)	↓***	↑^###^
Metenamine	Triazinane	2.76181	141.11347	141.11304	−3.068	C_6_H_12_N_4_	14.463	[M + H]^+^	141 (100), 85 (26.58), 98 (14.28), 71 (6.99),	↓**	↑
Docosahexaenoic Acid	Polyunsaturated fatty acids	7.10215	327.23295	327.23343	4.808	C_22_H_32_O_2_	17.838	[M − H]^−^	59 (100), 121 (5.99), 139 (4.60)	↑**	↓
9-Octadecenamide	Amides	2.8295	282.27914	282.27744	−4.27	C_18_H_35_NO	18.148	[M + H]^+^	69 (100), 55 (63.31), 57 (54.70), 81 (32.10), 71 (30.60), 67 (19.48)	↑***	↓^#^
Bovinic acid	Linoleic acids	1.3011	279.23295	279.23236	1.802	C_18_H_32_O_2_	18.805	[M − H]^−^	279 (100), 280 (22.90)	↑**	↓^##^
Stearic acid	Fatty acids	3.05534	283.26425	283.26379	2.235	C_18_H_36_O_2_	18.805	[M − H]^−^	283 (100), 284 (20.98)	↑***	↓^###^
trideca-6,8,10-trienoic acid	Fatty acids	1.79847	209.15360	209.15327	−2.899	C_13_H_20_O_2_	19.011	[M + H]^+^	89 (100), 103 (17.11), 53 (2.43)	↑**	↓^###^
13-Docosenamide	Amides	7.23946	338.34174	338.34085	−2.635	C_22_H_43_NO	19.562	[M + H]^+^	69 (100), 57 (77.64), 83 (61.17), 55 (68.67), 71 (37.89)	↑*	↓^###^

^*^p < 0.05, ^**^p < 0.01, ^***^p < 0.001, and ^****^p < 0.0001, B vs. Mod; ^#^*p* < 0.05, ^##^*p* < 0.01, and ^###^*p* < 0.001, HCat vs. Mod. The structures, molecular weights, and codes of differential metabolites were assigned according to the Human Metabolome Database.

### 3.6 Analysis of the correlation between metabolites, gut microbiota, and cytokines

Spearman correlation analysis was performed to examine associations among metabolites, gut microbial genera, and biochemical indicators (TC, TG, HDL-C, LDL-C, ALT, and AST), aiming to provide insight into the complex mechanisms by which catalpol exerts its therapeutic effects against HL.

**Figure 8:**
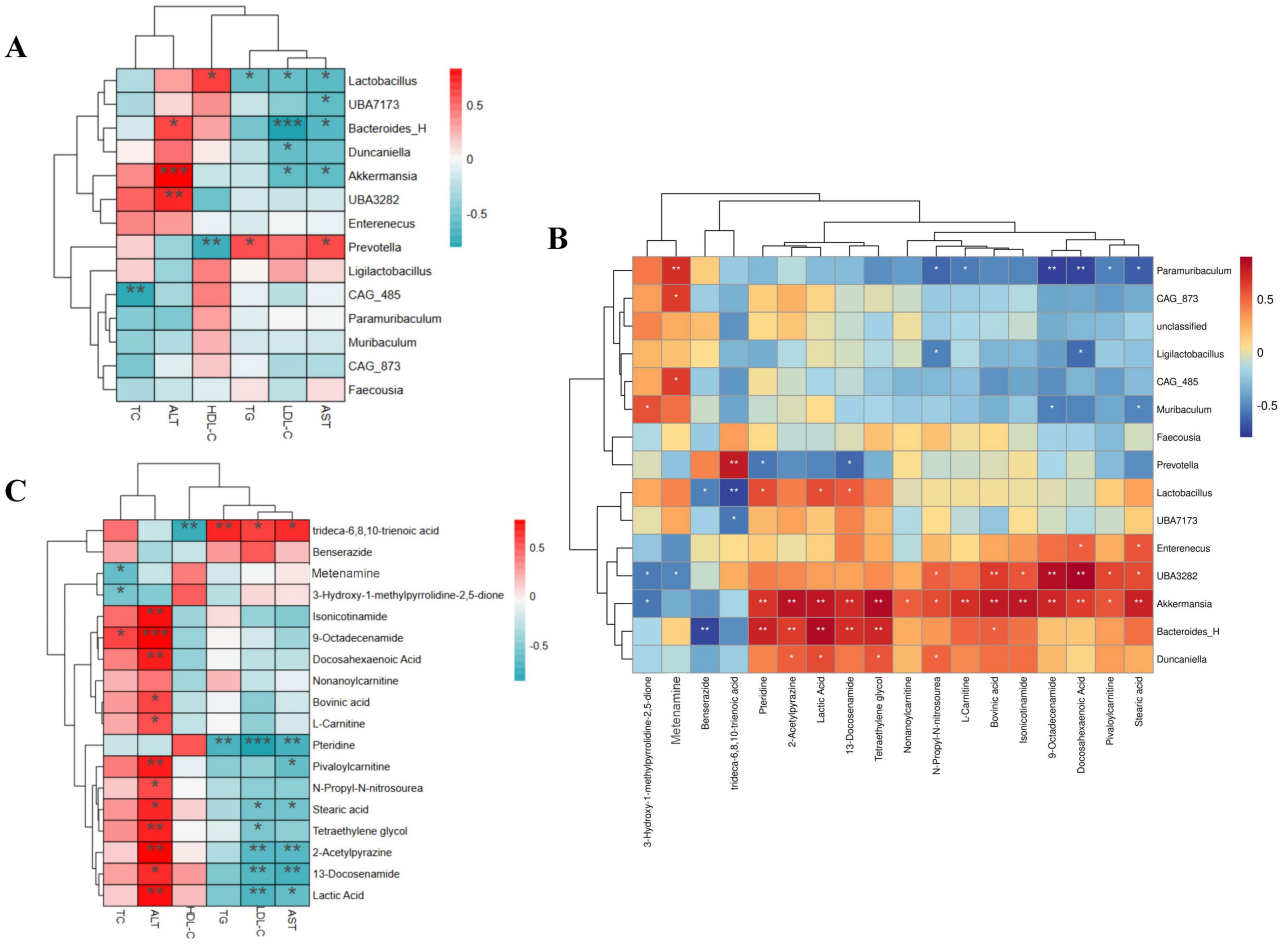
**(A)** Spearman correlation heat map analysis between gut microflora and HL-related cytokines. **(B)** Spearman correlation heat map analysis of the relationship between gut microbial flora and metabolites. **(C)** Spearman correlation heat map analysis between HL-related cytokines and metabolites.

The 15 most abundant bacterial genera were selected for correlation analysis. As shown in Figure 8A, the gut microbiota was closely related to serum biochemical changes. For example, *Bacteroides*_H and *Duncaniella* were negatively correlated with LDL-C (*p* < 0.05) and AST. Still, they showed positive correlations with ALT (*p* < 0.05), suggesting that these genera may not directly induce HL but could adversely affect liver function. *Prevotella* exhibited a negative correlation with HDL-C (*p* < 0.05), indicating that this genus may exacerbate HL-related damage. Interestingly, *Lactobacillus* was positively correlated with HDL-C (*p* < 0.05) and negatively correlated with other biochemical indicators, suggesting that it plays an ameliorative role in HL.

Further correlation analysis showed that significant associations existed between the gut microbiota and metabolites (Figure 8B). *Bacteroides*_H, *Duncaniella*, and *Akkermansia* were negatively correlated with benserazide and 3-hydroxy-1-methylpyrrolidine-2,5-dione, but positively correlated with the other metabolites. *Prevotella* showed a significant positive correlation with trideca-6,8,10-trienoic acid (*p* < 0.01) and negative correlations with all the other metabolites. Meanwhile, *Lactobacillus* was negatively correlated with benserazide and trideca-6,8,10-trienoic acid (*p* < 0.05), but positively correlated with pteridine, lactic acid, and 13-docosenamide. These results showed that distinct metabolite alterations correspond to specific bacterial genera and that high-dose catalpol may modulate the gut microbiota through metabolic regulation, leading to the amelioration of HL.

The correlations between metabolites and biochemical indicators are shown in Figure 8C. Twelve metabolites, including isonicotinamide, 9-octadecenamide, and docosahexaenoic acid, were positively correlated with ALT (*p* < 0.05), suggesting that these metabolites may exert hepatotoxic effects. Conversely, 2-acetylpyrazine, 13-docosenamide, and lactic acid were negatively correlated with LDL-C, indicating that while these metabolites may not exacerbate HL, they could nonetheless still negatively influence liver function. Trideca-6,8,10-trienoic acid was positively correlated with AST, LDL-C, and TG (*p* < 0.05), and negatively correlated with HDL-C (*p* < 0.01), implying that it both promotes HL and exerts adverse effects on hepatic function. Notably, pteridine showed significant negative correlations with TG, LDL-C, and AST (*p* < 0.05), and a positive correlation with HDL-C, suggesting that it may serve as a potential biomarker for the anti-HL activity of catalpol.

## 4 Discussion

As one of the systemic metabolic diseases, HL is associated with atherosclerosis, coronary heart disease, hypertension, and coronary heart diseases (Ballantyne et al., 2024; Toth, 2024). Based on previous studies, catalpol alleviated liver injury and further improved liver fibrosis by inhibiting aerobic glycolysis. In this experiment, we evaluated the specific function of catalpol on HFD-induced HL rats. HFD disrupted the homeostasis of lipids, leading to a large accumulation of lipids and causing damage to the liver of rats. Compared with normal rats, the levels of ALT and AST in HL rats were significantly changed, and the levels of TC, TG, and LDL-C in HL rats were higher than those in normal rats. Catalpol significantly reversed the above pathological trends. This result provides some evidence in support of the development of catalpol as a new lipid-lowering drug.

In this study, we found that catalpol significantly altered the gut microbiota composition while exerting its lipid-lowering effects. Changes in the gut microbiota of HL model rats and high-dose catalpol (HCat)-treated rats were analyzed by 16S rRNA sequencing. At the genus level, the HFD reduced the relative abundance of *Lactobacillus*, *Bifidobacterium*_388775, *Bacteroides*_H, *Parabacteroides*_B_862066, and *Duncaniella* (*p* < 0.05), while increasing that of *Prevotella* (*p* < 0.05). However, catalpol treatment reversed these HFD-induced alterations.

*Lactobacillus* and *Bifidobacterium*, which are acclaimed as key probiotics, can reduce intestinal cholesterol absorption. This reduction is achieved either by modulating the Niemann-Pick-like protein 1 (NPC1L1) or through processes such as entrainment, co-precipitation, and adhesion (Ma et al., 2025). Concurrently, *Lactobacillus* and *Bifidobacterium* secrete bile acid hydrolase. This enzyme catalyzes the conversion of cholesterol into bile acids, which promotes the transport of cholesterol from the blood to the liver. In the liver, cholesterol is converted into fecal sterols that cannot be absorbed and are excreted from the circulatory system, thereby reducing serum cholesterol levels. Moreover, *Lactobacillus and Bifidobacterium* can promote fatty acid β-oxidation and accelerate triglyceride breakdown by activating the peroxisome proliferator-activated receptor (PPAR) signaling pathway. Some *Bacteroides* (such as *Parabacteroides merdae*) are capable of degrading branched-chain amino acids (BCAAs) and producing the short-chain fatty acids (SCFAs). These metabolites contribute to the regulation of the host’s lipid metabolism and inflammatory response. Research has demonstrated that the supplementation of *Bacteroides* faecalis significantly reduces blood lipid levels in mice with HFD by inhibiting the mTORC1 signaling pathway within atherosclerotic plaques (Qiao et al., 2022). Similarly, ^13^C-inulin metagenomic and metabolomic analyses revealed that pentadecanoic acid is produced by *Parabacteroides* from inulin and that this acid exerts a protective effect against non-alcoholic fatty liver disease (NASH) in mice. Both *Parabacteroides* and pentadecanoic acid can restore intestinal barrier function in the NASH model, reduce plasma LPS levels, and suppress the expression of hepatic proinflammatory cytokines (Wei et al., 2023). Overall, these suggest that gut microbiota members could contribute to producing beneficial metabolites that inhibit lipid metabolic diseases.

**Figure 9:**
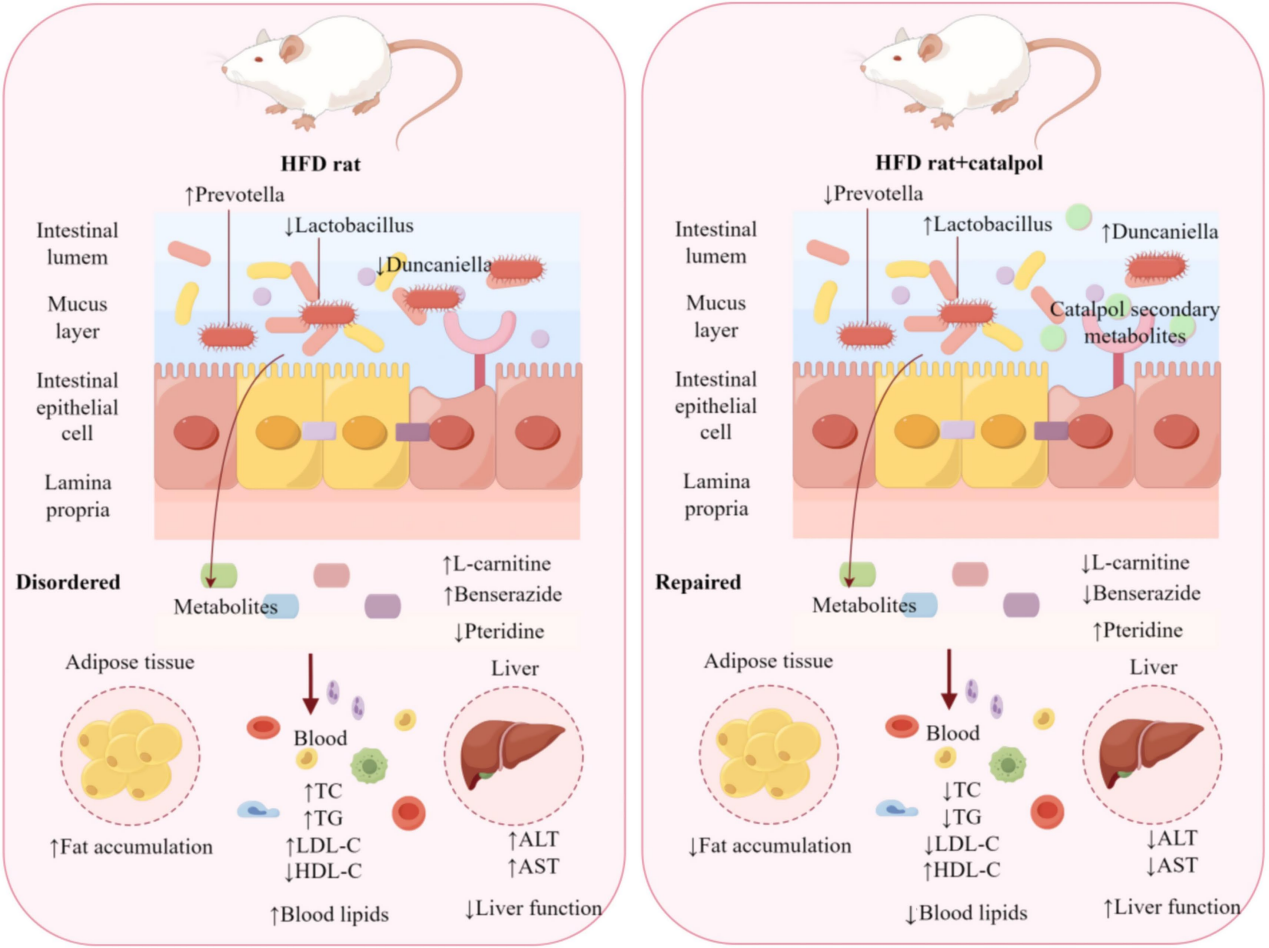
The mechanisms underlying the anti-HL effects of catalpol.

The metabolic behavior of natural compounds is closely linked to their therapeutic mechanisms, implying that catalpol metabolites may play an essential role in ameliorating HL. In this study, UHPLC-Q-Exactive Orbitrap MS was employed to identify and characterize catalpol metabolites. The metabolic pathways of catalpol were divided into two categories: the parent compound (catalpol) and its aglycone. Furthermore, three metabolites, M2, M15, and M18, were newly identified in this study, corresponding to the glycosylation, methylation, and hydroxylation products of catalpol, respectively. The multipathway metabolism of catalpol provides diverse molecular targets for HL intervention. Catalpol aglycone is first generated and subsequently transformed into a series of metabolites through phase I reactions, including hydroxylation, dihydroxylation, and hydrogenation. These metabolites are associated with HL. For example, dihydrotanshinone I, a hydrogenation product, acts as a PPARα agonist and contributes to the treatment of NASH (Shou et al., 2024). Most catalpol metabolites (such as M1 and M2) were detected in fecal samples, highlighting the key contribution of the gut microbiota to catalpol metabolism and its lipid-lowering effects. The gut microbiota-derived metabolites of catalpol exhibit pharmacological activities. Following intestinal absorption, these metabolites enter the systemic circulation and contribute to lowering blood lipid levels (Figure 9).

The synthesis and degradation of endogenous metabolites can lead to metabolic disorders. Using UHPLC-Q-Exactive-Orbitrap-MS-based non-targeted metabolomics, we have preliminarily explored the mechanism underlying the anti-HL therapeutic effect of catalpol (Su et al., 2024; Wang et al., 2024). Sixteen metabolites were found to be significantly upregulated (*p* < 0.05) while two were significantly downregulated (*p* < 0.05) in the Mod group relative to the Blank group. However, treatment with high-dose catalpol markedly reversed these alterations. Most of the identified metabolites were fatty acids and carnitines. The inhibition of carnitine metabolism under HFD conditions leads to affected fatty acid oxidation, increased oxidative stress, and lipid accumulation (Zhao et al., 2022). The metabolic adjustments induced by catalpol indicate that its lipid-lowering effect may involve restoring metabolic processes.

Finally, we examined the correlations among gut microbiota, endogenous metabolites, and serum cytokines. *Lactobacillus* showed a significant positive correlation with HDL-C (*p* < 0.05) and a negative correlation with other biochemical indicators, implying that *Lactobacillus* may play a key role in the lipid-lowering effect of catalpol. Moreover, pteridine was significantly negatively correlated with TG, LDL-C, and AST (*p* < 0.05), and positively correlated with HDL-C. These findings suggest that pteridine may serve as a potential biomarker for the anti-HL activity of catalpol. Pteridine is a precursor of tetrahydrobiopterin (BH_4_), which is known to improve vascular function and modulate lipid metabolism through the activation of nitric oxide synthase. However, the relationship between pteridine and *Lactobacillus* remains unclear and warrants further investigation.

In conclusion, we established a rat model of HFD-induced HL to investigate the therapeutic effects of catalpol. By integrating 16S rRNA sequencing with non-targeted metabolomics, we explored the internal regulatory mechanisms by which catalpol influences gut microbiota composition and the metabolic profile of HL model rats. Our findings demonstrate that HL is associated with gut dysbiosis and alterations in plasma metabolite secretion, both of which are effectively reversed by catalpol treatment. Overall, catalpol enhances lipid metabolism, including its degradation, by increasing the abundance of lipid transport-associated genera such as *Bacteroides* and *Lactobacillus*, while reducing that of inflammation-related genera such as *Prevotella*. Using UHPLC-Q-Exactive Orbitrap MS, we further analyzed the metabolites of catalpol in plasma, urine, and feces to characterize its metabolism. This is the first study to investigate the correlations between the gut microbiota and these metabolites from the perspective of endogenous metabolites. It provides novel insights into the mechanism by which catalpol exerts its anti-HL effects. Further studies are needed to verify the specific roles of the dominant gut microbial taxa and metabolites in HL. Our analysis reveals the potential mechanism underlying the ameliorative effects of catalpol on HL, thereby providing a promising basis for its development as a therapeutic agent for lipid metabolic disorders.

## Data Availability

The datasets have been uploaded to the publicly accessible databases below: 1. Metabolite and metabolome profiling data: Source data have been deposited to the EMBL-EBI MetaboLights database with the identifier MTBLS13187. 2. 16S rRNA data: Raw data have been deposited to National Center for Biotechnology Information (NCBI) under the BioProject number PRJNA1347082.

## Footnotes

^1^ https://www.bioincloud.tech/
