# Supplementary material for: Catalpol—a compound from Rehmannia glutinosa can improve hyperlipidemia by modulating gut microbiota and endogenous metabolic pathways
Source: Front Microbiol. 2025 Nov 11;16:1689778. doi: 10.3389/fmicb.2025.1689778 (PMC12644921; doi:10.3389/fmicb.2025.1689778)
Supplement: Supplementary file 1 [file Supplementary_file_1.zip › Sumpplement/Sumpplement Figure S1.docx]

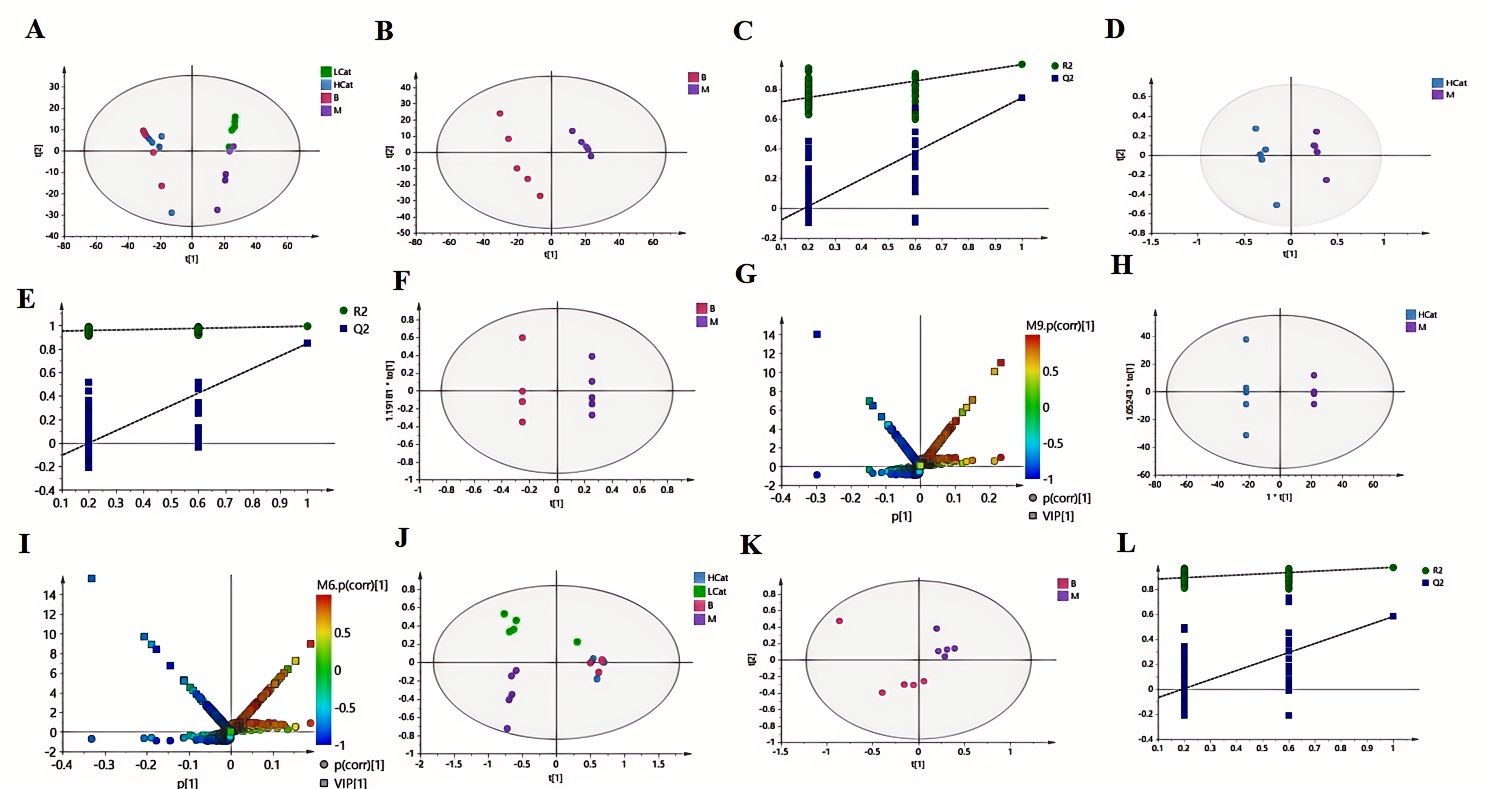

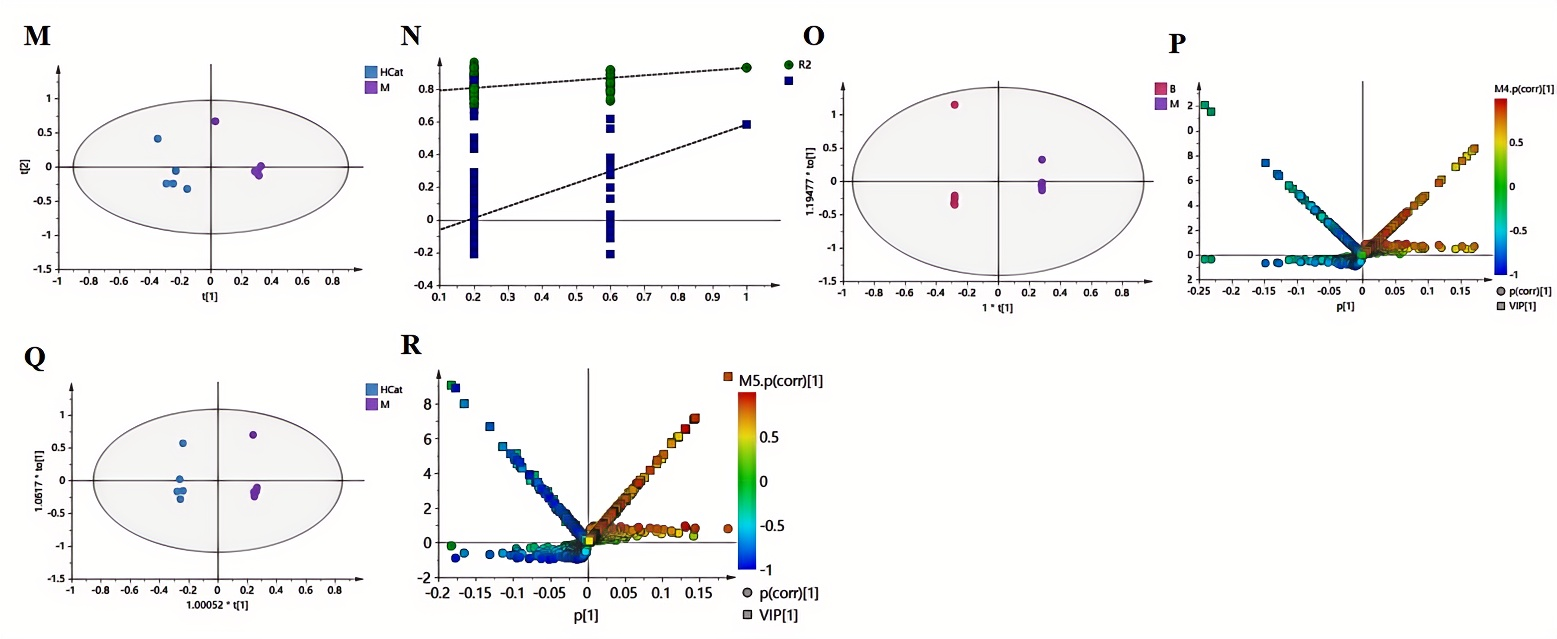


**Sumpplement Figure S1.** **Effect of catalpol on metabolic parameters in high-fat rats and related analysis**. B: Blank group; M: high-fat diet group; HCat: catalpol high dose treatment group; LCat: low-dose catalpol treatment group. (A) Positive PCA score plot. (B-C) B VS. M Positive PLS-DA score plot; B VS. M Positive PLS-DA model replacement test chart. (D-E) M VS. HCat Positive PLS-DA score plot; M VS. HCat Positive PLS-DA model replacement test chart. (F-G) B VS. M Positive OPLS-DA score plot; B VS. M Positive S-V plots. (H-I) M VS. HCat Positive OPLS-DA score plot; M VS. HCat Positive S-V plots. (J) Negative PCA score plot. (K-L) B VS. M Negative PLS-DA score plot; B VS. M Negative PLS-DA model replacement test chart. (M-N) M VS. HCat Negative PLS-DA score plot; M VS. HCat Negative PLS-DA model replacement test chart. (O-P) B VS. M Negative OPLS-DA score plot; B VS. M Negative S-V plots. (Q-R) M VS. HCat Negative OPLS-DA score plot ; M VS. HCat Negative S-V plo
