# Supplementary material for: Catalpol—a compound from Rehmannia glutinosa can improve hyperlipidemia by modulating gut microbiota and endogenous metabolic pathways
Source: Front Microbiol. 2025 Nov 11;16:1689778. doi: 10.3389/fmicb.2025.1689778 (PMC12644921; doi:10.3389/fmicb.2025.1689778)
Supplement: Supplementary file 1 [file Supplementary_file_1.zip › Sumpplement/Supplement table S1.docx]

**Supplement table S1**

| Maintenance feed (Blank group) | | High-fat feed (High fat diet group: Mod, LCat, HCat) | | |
| --- | --- | --- | --- | --- |
| Name | Proportion | Name | Proportion | |
| Crude protein | 22.2% | Crude protein | 18.0% | Maintenance feed (65.0%) |
| Coarse ash | 6.7% | Coarse ash | 8.0% |  |
| Crude fiber | 4.5% | Crude fiber | 5.0%% |  |
| Crude fat | 5.1% | Crude fat | 4.0% |  |
| Calcium | 1.01% | Calcium | 1.0~1.8% |  |
| Total phosphorus | 0.91% | Total phosphorus | 0.60%~1.20% |  |
| Water | 10.5% | Water | 10.00% |  |
|  |  | Pig fat | 15.0% | |
|  |  | Cholesterol | 5.0% | |
|  |  | Powdered yolk | 10.0% | |
|  |  | Sodium bile acid | 5.0% | |
